# Rapid Spread of the SARS-CoV-2 Variant of Concern 202012/01 in Southern Italy (December 2020–March 2021)

**DOI:** 10.3390/ijerph18094766

**Published:** 2021-04-29

**Authors:** Daniela Loconsole, Francesca Centrone, Caterina Morcavallo, Silvia Campanella, Anna Sallustio, Marisa Accogli, Francesca Fortunato, Antonio Parisi, Maria Chironna

**Affiliations:** 1 Department of Biomedical Sciences and Human Oncology-Hygiene Section, University of Bari, 70124 Bari, Italy; daniela.loconsole@uniba.it (D.L.); francesca.centrone.fc@gmail.com (F.C.); caterinamorcavallo@gmail.com (C.M.); campanella.silvia@libero.it (S.C.); accoisa@gmail.com (M.A.); 2 Hygiene Unit, Azienda Ospedaliero-Universitaria Consorziale Policlinico di Bari, 70124 Bari, Italy; annasallustio@libero.it; 3 Department of Medical and Surgical Sciences, University of Foggia, 71122 Foggia, Italy; francesca.fortunato@unifg.it; 4 Istituto Zooprofilattico Sperimentale della Puglia e della Basilicata, 71121 Foggia, Italy; antonio.parisi@izspb.it

**Keywords:** surveillance, VOC 202012/01-B.1.1.7 lineage SARS-CoV-2, variants of concern, COVID-19, Italy

## Abstract

Epidemiological and virological studies have revealed that SARS-CoV-2 variants of concern (VOCs) are emerging globally, including in Europe. The aim of this study was to evaluate the spread of B.1.1.7-lineage SARS-CoV-2 in southern Italy from December 2020–March 2021 through the detection of the S gene target failure (SGTF), which could be considered a robust proxy of VOC B.1.1.7. SGTF was assessed on 3075 samples from week 52/2020 to week 10/2021. A subset of positive samples identified in the Apulia region during the study period was subjected to whole-genome sequencing (WGS). A descriptive and statistical analysis of the demographic and clinical characteristics of cases according to SGTF status was performed. Overall, 20.2% of samples showed SGTF; 155 strains were confirmed as VOC 202012/01 by WGS. The proportion of SGTF-positive samples rapidly increased over time, reaching 69.2% in week 10/2021. SGTF-positive cases were more likely to be symptomatic and to result in hospitalization (*p* < 0.0001). Despite the implementation of large-scale non-pharmaceutical interventions (NPIs), such as the closure of schools and local lockdowns, a rapid spread of VOC 202012/01 was observed in southern Italy. Strengthened NPIs and rapid vaccine deployment, first among priority groups and then among the general population, are crucial both to contain the spread of VOC 202012/01 and to flatten the curve of the third wave.

## 1. Introduction

A new variant of SARS-CoV-2 was first identified in southeast England in early October 2020. Epidemiological and virological studies revealed that the strain belonged to a new single phylogenetic cluster of lineage B.1.1.7. In December 2020, Public Health England declared this new strain a variant of concern (VOC 202012/01, also termed the UK variant) [1]. In addition, at the end of December 2020, another SARS-CoV-2 variant was identified in South Africa in samples collected in October 2020 and designated as 501.V2. This variant became the dominant form of the virus in that country [2]. Another SARS-CoV-2 variant was identified in Brazil (mostly in the Amazonas state) and designated the P.1 variant [3]. All of these variants are considered to be of concern because of the presence of mutations leading to increased transmissibility and rapid epidemiological changes. The latest risk assessment of the European Center for Disease Control (ECDC) states that these variants are more easily transmitted and could cause more severe disease [4]. However, the real impact of these variants on clinical presentation and patient outcomes, and on the efficacy of the available vaccines, is still poorly understood. Existing licensed COVID-19 vaccines may be only partially effective or significantly less effective against a variant. For this reason, on 15 February 2021, the ECDC reported that the risk associated with the spread of the SARS-CoV-2 VOCs in the EU/EEA was high/very high for the overall population and very high for vulnerable individuals [4]. The rapid spread of VOCs in Europe is causing a rapid increase in the number of cases, thus putting significant pressure on the healthcare system. The mutations observed in the new variants are related to the receptor binding site and other surface structures [5]. The most concerning mutation is E484K because a reduction in the neutralization activity of polyclonal serum antibodies has been attributed to this mutation [4].

VOC 202012/01 showed an increased estimated transmissibility of up to 70% [5]. A recent study by Davies et al. estimated the overall hazard of death associated with B.1.1.7-lineage SARS-CoV-2 infection as 61% or higher, suggesting that this VOC is not only associated with greater transmissibility but also with more severe illness [6]. At the time of writing, the B.1.1.7 variant has been detected in all EU/EEA countries that have any significant detection capability [4]. In Italy, a flash survey conducted in February 2021 suggested that the prevalence of this variant increased from 17.8% to 54.0% in 2 weeks, increasing to 86.7% in March 2021 [7,8]. VOC 202012/01 is characterized by 14 genetic mutations, eight of which are located in the portion of the genome encoding the spike protein [9]. One of the characteristic mutations is the Δ69/70 deletion, which impairs the detection of the S gene using common commercial real-time PCR kits. In the UK, Israel, and Portugal, a strong association between this S gene target failure (SGTF) and the circulation of the B.1.1.7 variant was reported [5,10,11,12]. This association could vary in other countries because other variants could also produce S gene-negative results. Due to its high prevalence in the UK, SGTF has been used as a proxy to track the circulation of B.1.1.7-lineage VOC [13]. In the Apulia region (southern Italy), VOC 202012/01 was first identified at the end of December 2020 [14]. In three flash surveys conducted to estimate the prevalence of VOC 202012/01 in Italy, all of the samples collected in the Apulia region and showing SGTF were confirmed as B.1.1.7-lineage strains by whole-genome sequencing [7,8], suggesting that S gene dropout could be considered a robust proxy for assessing the circulation of the UK variant.

In the present study, we investigated the proportion of SGTF to gain insight into the spread of the B.1.1.7 variant in the Apulia region (southern Italy) from December 2020 to March 2021, and to assess the clinical characteristics of the infected subjects. A subset of positive samples identified in the Apulia region during the study period was subjected to whole-genome sequencing to confirm the presence of the B.1.1.7-lineage SARS-CoV-2 variant.

## 2. Materials and Methods

### 2.1. Epidemiological Surveillance

The COVID-19 national surveillance system in Italy includes integrated epidemiological and microbiological surveillance. All cases of suspected SARS-CoV-2 infection are investigated at the local level and a network of regional laboratories processes respiratory samples from all suspected SARS-CoV-2 cases. All epidemiological information and molecular data from confirmed SARS-CoV-2 cases are uploaded to an online national surveillance platform [15].

### 2.2. Molecular Test for SARS-CoV-2 Infection and Whole-Genome Sequencing

For the purpose of this study, we utilized 3075 samples collected from new cases of SARS-CoV-2 infection identified every Tuesday from week 52/2020 (22 December 2020) to week 10/2021 (9 March 2021) at the Laboratory of Molecular Epidemiology and Public Health of the Hygiene Unit (A.O.U.C. Policlinico Bari, Italy), which is the coordinator of the Regional Laboratory Network for SARS-CoV-2 diagnosis in the Apulia region. The laboratory processes an average of 3000 nasopharyngeal swabs per day, collected from both hospitalized and non-hospitalized patients, covering 60% of the province of Bari (1,230,205 inhabitants). RNA was extracted using the MagMAX Viral/Pathogen Nucleic Acid Isolation Kit (Thermo Fisher Scientific, Waltham, MA, USA) and the molecular test was performed using TaqPath RT-PCR COVID-19 Assay, a three-target commercial multiplex real-time PCR assay based on the identification of the N, ORF1ab, and S genes (Thermo Fisher Scientific) [5]. The extracted RNA (10 μL) was transferred to 96 well PCR plates containing 6.25 μL of TaqPathTM 1-step Multiplex Master Mix (No ROX), 1.25 μL of COVID-19 real time PCR Assay Multiplex, and 7.5 μL of nuclease free water, and subjected to one-step RT-PCR (Thermo-Fisher TaqPath COVID-19 assay kit) on a QuantStudio™ 5 Real Time PCR System (Applied Biosystems).

To support the accuracy of S gene target failure (SGTF) as a proxy for B.1.1.7 and the estimated frequency of VOC 202012/01 in the Apulia region, 155 SGTF-positive samples were selected to meet the regional requirement for the molecular surveillance and the national requirement to estimate the diffusion of the VOCs in Italy [7], and subjected to sequencing, which was performed as previously described [14].

### 2.3. Data Analysis

Data analysis was performed on information collected from the COVID-19 online national surveillance platform. For each patient, the worst clinical condition registered in the database was considered. Statistical analyses were performed with STATA 12.0 software. The 95% confidence intervals are reported for proportions. Chi-squared or Fisher’s exact tests were used to compare proportions between SGTF-positive cases and SGTF-negative cases. A *p*-value of <0.05 was considered significant. Clinical cases were classified according to the Italian COVID-19 surveillance system [15] as asymptomatic, mild infection (fever < 38 °C, cough, malaise, gastrointestinal symptoms without fever), moderate infection (fever ≥38 °C, upper respiratory symptoms, anosmia/ageusia, gastrointestinal symptoms), or severe infection (fever ≥38 °C with dyspnea and lower respiratory tract infection). A multivariate logistic regression model was used to explore associations between clinical status, hospitalization, and SGTF status.

### 2.4. Ethical Statement

Ethical approval was not required because the activities described were conducted as part of the legislated mandate of the Health Promotion and Public Health Department of the Apulia region (Italy). All procedures were carried out in accordance with the Declaration of Helsinki, as revised in 2013, for research involving human subjects.

## 3. Results

**Figure 1:**
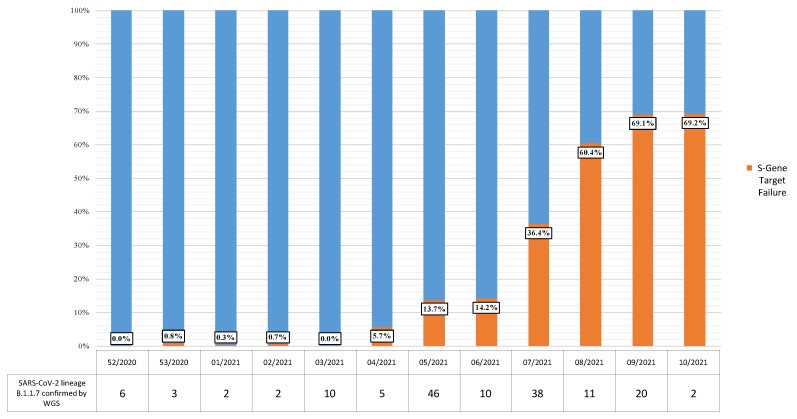
Distribution (%) of S gene target failure (SGTF)-positive cases among new SARS-CoV-2 infections identified from week 52/2020 to week 10/2021 in the Apulia region of Italy, and SARS-CoV-2 strains confirmed as VOC 202012/01-B.1.1.7 lineage by whole genome sequencing, by week.

Overall, 3075 new SARS-CoV-2 infections in subjects residing in the province of Bari were considered in the study. The median age was 43 years, and 1469 patients (47.8%) were male. Among newly diagnosed cases, 20.2% (621/3075) showed SGTF and were assumed to be positive for B.1.1.7-lineage SARS-Cov-2 VOC 202012/01. The proportion of samples showing SGTF rapidly increased over time, from 0% in week 52/2020 to 69.2% in week 10/2021 (Figure 1). Specifically, 0/302 cases were SGTF-positive in week 52/2020, 3/397 in week 53/2020, 1/286 in week 1/2021, 2/289 in week 2/2021, 0/260 in week 3/2021, 12/211 in week 4/2021, 27/197 in week 5/2021, 33/233 in week 6/2021, 67/184 in week 7/2021, 131/217 in week 8/2021, 181/262 in week 9/2021, and 164/237 in week 10/2021.

The demographic and clinical characteristics of the patients according to SGTF status are reported in Table 1. There were no significant differences in sex or age distribution between the two groups except for the 17–35-year-old age group, which was more highly represented among SGTF-negative cases (*p* = 0.04). Compared with SGTF-negative cases, SGTF-positive cases were more likely to be symptomatic (*p* < 0.0001), but the correlation with the severity of illness was not statistically significant in the sample analyzed in the study. However, SGTF-positive cases were more likely to result in hospitalization (*p* < 0.0001). Specifically, by multivariate logistic regression analysis, the SGTF-positive group showed an odds ratio of 3.44 (confidence interval (CI), 1.76–6.75) for hospitalization, confirming that the presence of SGTF is related to a worse evolution of the infection. There was no difference in the number of deaths in the two groups.

### Sequence Analyses

Whole-genome sequencing was performed on 155 SGTF-positive samples collected during the study period. The sequences encompassed the entire genome. Genomic analysis showed that all the strains were B.1.1.7-lineage SARS-CoV-2. The whole-genome sequences were deposited in the GISAID database (Supplementary Materials). In the Apulia region, from December 2020 to March 2021, 279 strains were sequenced for the regional molecular surveillance (all sequences are available in the GISAID database). Of these strains, only 5/279 (1.8%) showing the SGTF were confirmed to be strains other than B.1.1.7 by WGS. No other SARS-CoV-2 variants characterized by a mutation in the position 69–70 have been yet identified in the Apulia region.

## 4. Discussion

The present study shows that during the period of December 2020–March 2021, a rapid increase in the spread of VOC 202012/01 SARS-CoV-2 was observed in southern Italy. The circulation of VOC 202012/01 in the Apulia region rapidly increased during each week of observation, as reported in other countries [12]. Based on these data, we can speculate that, at the time of writing, the UK variant has almost completely replaced the circulation of wild-type SARS-CoV-2 in our region.

In countries with a high prevalence of VOC 202012/01, SGTF has been used as a robust proxy for monitoring the diffusion of B.1.1.7-lineage SARS-CoV-2 [10,12]. In the Apulia region, the B.1.1.7-lineage strain was identified in 100% of the SGTF-positive samples of the flash surveys carried out in February (45/45 and 28/28 samples) and March 2021 (117/117 samples) [7,8]. Overall, 98.2% of the samples showing the SGTF and selected for the regional and national surveillance that were subjected to whole-genome sequencing were confirmed as VOC 202012/01. Moreover, no other SARS-CoV-2 variants of concern characterized by the ∆69–70 have been yet identified in the Apulia region. Based on these data, we can speculate that, in the Apulia region, SGTF could be considered to be a robust proxy to identify B.1.1.7-lineage strains and, therefore, to estimate the circulation of these VOCs.

Compared with SGTF-negative patients, there was no significant difference in the age distribution in the SGTF-positive group, except for the 17–35-year-old age group, which was significantly more represented in the SGTF-negative group. These data are largely consistent with those reported for Portugal [12], where no differences in age distribution among people infected with B.1.1.7-lineage SARS-CoV-2 and wild-type SARS-CoV-2 were identified. Infection with VOC 202012/01 was also associated with an increased risk of symptomatic disease and hospitalization in our study. A more severe illness in the presence of B.1.1.7-lineage infection was demonstrated in the UK, whereas the correlation with the severe clinical state of illness was not statistically significant in the sample analyzed in the present study [6]. In our study, there was no significant increase in the risk of death in subjects infected with the UK variant. By contrast, in a recent study, Davies et al. estimated a substantial increase in the risk of death among people infected with B.1.1.7-lineage SARS-CoV-2. This increased risk occurred specifically in older age groups, whereas the risk of death following a positive test was estimated to be below 1% in most individuals younger than 70 [6]. Challen et al. also reported that infection with the UK variant caused additional mortality compared with previously circulating variants [10]. The higher viral load in SGTF-positive patients, which may be linked to the mutations in B.1.1.7-lineage SARS-CoV-2, has been hypothesized as a possible explanation for the increased mortality [10].

Despite the implementation of large-scale non-pharmaceutical interventions (NPIs), such as the closure of schools and local lockdowns, a novel wave of viral spread has been registered in Italy in the past several weeks [16]. This so-called third wave is increasing pressure on hospital systems and resulting in delays in treatment, stressing the national healthcare service in Italy. Recently, increased transmission of SARS-CoV-2 has been documented in the Apulia region, likely related to the diffusion of the VOC 202012/01. To facilitate the monitoring and the management of cases, an interactive dashboard to signal SGTF-positive cases to the Local Health Authority was implemented [17]. These real-time data are useful to support timely public health decisions by early identification of suspected VOC 202012/01 cases. Moreover, these data showed an immediate impact, because they triggered the political decision to strengthen non-pharmaceutical interventions, such as school closure, in the Apulia region before school closures were implemented nationally.

At this point in the pandemic, the application of strengthened NPIs and rapid vaccine deployment, first among priority groups and then among the general population, are crucial both to contain the spread of VOC 202012/01 and to flatten the curve of the third wave. However, the recent decision to halt the administration of the AstraZeneca vaccine in Italy on 15 March 2021 [18] might slow the containment of the pandemic in Italy by delaying the vaccination campaign. This decision could also decrease patient confidence in vaccine efficacy, although the ban was lifted on 19 March 2021 [19]. Moreover, the spread of new variants of SARS-CoV-2 raises concerns about these variants as a possible cause of re-infections and post-vaccination infections because the current licensed vaccines might have reduced efficacy against these strains [4].

## 5. Conclusions

Urgent measures to prevent the spread of novel variants with selective advantages are promptly required [20]. NPIs and strict epidemiological and molecular surveillance remain the cornerstones of the response to the COVID-19 pandemic [4]. Further studies, including a genetic comparison of SARS-CoV-2 strains, will be necessary to better understand the spread of SARS-CoV-2, particularly in light of the concerning spread of SARS-CoV-2 VOCs.

## Figures and Tables

**Table 1:** Demographic and clinical characteristics of SGTF-positive and SGTF-negative cases identified from week 52/2020 to week 10/2021 in the Apulia region of Italy.

Characteristics	SGTF-Positive	SGTF-Negative	*p*-Value
Sex			
Male	313 (50.4%)	1156 (47.2%)	0.14
Female	308 (49.6%)	1298 (53.0%)	0.14
Age groups (%)			
0–4 yrs	21 (3.38%)	70 (2.85%)	0.57
5–16 yrs	75 (12.08%)	268 (10.92%)	0.45
17–35 yrs	144 (23.19%)	670 (27.30%)	0.04
36–65 yrs	272 (43.80%)	1048 (42.71%)	0.65
>65 yrs	109 (17.55%)	398 (16.22%)	0.45
Symptomatic infection	321 (51.7%)	963 (39.3%)	<0.0001
Mild	214 (66.7%)	625 (64.9%)	0.61
Moderate	86 (26.8%)	289 (30.0%)	0.30
Severe	21 (6.5%)	49 (5.1%)	0.39
Asymptomatic infection	300 (48.3%)	1491 (60.7%)	<0.0001
Hospitalization	35 (5.6%)	69 (2.8%)	<0.0001
Death	4 (0.6%)	23 (0.9%)	0.64

## Data Availability

Data are available on request from the corresponding author.

## Supplementary Materials

The following are available online at https://www.mdpi.com/article/10.3390/ijerph18094766/s1, File S1. Whole-genome sequences in GISAID database.
