# Supplementary material for: Rapid Spread of the SARS-CoV-2 Variant of Concern 202012/01 in Southern Italy (December 2020–March 2021)
Source: Int J Environ Res Public Health. 2021 Apr 29;18(9):4766. doi: 10.3390/ijerph18094766 (PMC8125211; doi:10.3390/ijerph18094766)
Supplement: Supplementary file 1 [file ijerph-18-04766-s001.zip › ijerph-1182192-supplementary.pdf]

Accession ID	Collection date	Submission date	Location
EPI_ISL_1000969	02/01/21	15/02/21	Europe / Italy / Apulia / Bari
EPI_ISL_1000970	09/01/21	15/02/21	Europe / Italy / Apulia / Taranto / Ginosa
EPI_ISL_1007642	21/01/21	16/02/21	Europe / Italy / Apulia
EPI_ISL_1008663	03/02/21	16/02/21	Europe / Italy / Apulia / Bari / Altamura
EPI_ISL_1008664	21/01/21	16/02/21	Europe / Italy / Apulia / Lecce
EPI_ISL_1008665	03/02/21	16/02/21	Europe / Italy / Apulia / Bari / Modugno
EPI_ISL_1008666	02/02/21	16/02/21	Europe / Italy / Apulia / Bari / Bitetto
EPI_ISL_1008667	02/02/21	16/02/21	Europe / Italy / Apulia / Bari / Bitetto
EPI_ISL_1008668	03/02/21	16/02/21	Europe / Italy / Apulia / Bari / Bitetto
EPI_ISL_1008669	02/02/21	16/02/21	Europe / Italy / Apulia / Bari / Bitetto
EPI_ISL_1008670	02/02/21	16/02/21	Europe / Italy / Apulia / Bari / Bitetto
EPI_ISL_1008671	03/02/21	16/02/21	Europe / Italy / Apulia / Bari
EPI_ISL_1008672	01/02/21	16/02/21	Europe / Italy / Apulia / Bari / Santeramo in Colle
EPI_ISL_1008673	03/02/21	16/02/21	Europe / Italy / Apulia / Bari
EPI_ISL_1008674	01/02/21	16/02/21	Europe / Italy / Apulia / Bari / Santeramo in Colle
EPI_ISL_1008675	03/02/21	16/02/21	Europe / Italy / Apulia / Brindisi / Carovigno
EPI_ISL_1008676	03/02/21	16/02/21	Europe / Italy / Apulia / Brindisi / Carovigno
EPI_ISL_1008677	04/02/21	16/02/21	Europe / Italy / Apulia / Brindisi / Fasano
EPI_ISL_1008678	26/01/21	16/02/21	Europe / Italy / Apulia / Brindisi / Fasano
EPI_ISL_1008679	03/02/21	16/02/21	Europe / Italy / Apulia / Bari / Molfetta
EPI_ISL_1008680	04/02/21	16/02/21	Europe / Italy / Apulia / Bari
EPI_ISL_1008681	03/02/21	16/02/21	Europe / Italy / Apulia / Bari / Molfetta
EPI_ISL_1008682	03/02/21	16/02/21	Europe / Italy / Apulia / Bari / Bitritto
EPI_ISL_1008683	03/02/21	16/02/21	Europe / Italy / Apulia / Bari / Molfetta
EPI_ISL_1008684	03/02/21	16/02/21	Europe / Italy / Apulia / Bari / Modugno
EPI_ISL_1008685	04/02/21	16/02/21	Europe / Italy / Apulia / Bari / Sannicandro di Bari
EPI_ISL_1008686	03/02/21	16/02/21	Europe / Italy / Apulia / Bari / Bitetto
EPI_ISL_1008687	01/02/21	16/02/21	Europe / Italy / Apulia / Bari / Bitetto
EPI_ISL_1008688	03/02/21	16/02/21	Europe / Italy / Apulia / Bari
EPI_ISL_1008689	03/02/21	16/02/21	Europe / Italy / Apulia / Bari
EPI_ISL_1008690	01/02/21	16/02/21	Europe / Italy / Apulia / Bari / Santeramo in Colle
EPI_ISL_1008691	03/02/21	16/02/21	Europe / Italy / Apulia / Bari / Santeramo in Colle
EPI_ISL_1008692	01/02/21	16/02/21	Europe / Italy / Apulia / Bari / Santeramo in Colle
EPI_ISL_1008693	03/02/21	16/02/21	Europe / Italy / Apulia / Brindisi / Fasano
EPI_ISL_1008694	01/02/21	16/02/21	Europe / Italy / Apulia / Bari / Santeramo in Colle
EPI_ISL_1008695	03/02/21	16/02/21	Europe / Italy / Apulia / Bari
EPI_ISL_1008696	03/02/21	16/02/21	Europe / Italy / Apulia / Bari / Modugno
EPI_ISL_1008697	03/02/21	16/02/21	Europe / Italy / Apulia / Bari / Modugno
EPI_ISL_1008698	03/02/21	16/02/21	Europe / Italy / Apulia / Bari / Modugno
EPI_ISL_1008699	04/02/21	16/02/21	Europe / Italy / Apulia / Brindisi / Fasano
EPI_ISL_1008700	21/01/21	16/02/21	Europe / Italy / Apulia / Lecce
EPI_ISL_1008701	04/02/21	16/02/21	Europe / Italy / Apulia / Taranto / Massafra
EPI_ISL_1008702	01/02/21	16/02/21	Europe / Italy / Apulia / Bari / Palo del Colle

EPI_ISL_1008703	15/01/21	16/02/21	Europe / Italy / Apulia / Brindisi
EPI_ISL_1112223	08/02/21	01/03/21	Europe / Italy / Apulia / Lecce / Martano
EPI_ISL_1112224	08/02/21	01/03/21	Europe / Italy / Apulia / Lecce / Veglie
EPI_ISL_1112225	06/02/21	01/03/21	Europe / Italy / Apulia / Lecce / Cannole
EPI_ISL_1112226	11/02/21	01/03/21	Europe / Italy / Apulia / Lecce / Lecce
EPI_ISL_1112227	15/01/21	01/03/21	Europe / Italy / Apulia / Lecce / Cavallino
EPI_ISL_1112239	19/02/21	01/03/21	Europe / Italy / Apulia / Bari / Bari
EPI_ISL_1112241	18/02/21	01/03/21	Europe / Italy / Apulia / Bari / Corato
EPI_ISL_1112243	18/02/21	01/03/21	Europe / Italy / Apulia / Bari / Bari
EPI_ISL_1112245	18/02/21	01/03/21	Europe / Italy / Apulia / Bari / Gravina di Puglia
EPI_ISL_1112246	17/02/21	01/03/21	Europe / Italy / Apulia / Bari / Bari
EPI_ISL_1112247	11/02/21	01/03/21	Europe / Italy / Apulia / Bari / Acquaviva delle Fonti
EPI_ISL_1112248	09/02/21	01/03/21	Europe / Italy / Apulia / Bari / Altamura
EPI_ISL_1112250	18/02/21	01/03/21	Europe / Italy / Apulia / Bari / Capurso
EPI_ISL_1112252	18/02/21	01/03/21	Europe / Italy / Apulia / Bari / Altamura
EPI_ISL_1112253	12/02/21	01/03/21	Europe / Italy / Apulia / Bari / Adelfia
EPI_ISL_1112254	17/02/21	01/03/21	Europe / Italy / Apulia / Bari / Capurso
EPI_ISL_1112258	18/02/21	01/03/21	Europe / Italy / Apulia / Brindisi / Torre Santa Susanna
EPI_ISL_1112260	18/02/21	01/03/21	Europe / Italy / Apulia / Brindisi / Fasano
EPI_ISL_1112263	18/02/21	01/03/21	Europe / Italy / Apulia / Brindisi / Brindisi
EPI_ISL_1112265	16/02/21	01/03/21	Europe / Italy / Apulia / Barletta-Andria-Trani / Canosa di Puglia
EPI_ISL_1112266	17/02/21	01/03/21	Europe / Italy / Apulia / Barletta-Andria-Trani / Canosa di Puglia
EPI_ISL_1112267	18/02/21	01/03/21	Europe / Italy / Apulia / Barletta-Andria-Trani / Canosa di Puglia
EPI_ISL_1112273	17/02/21	01/03/21	Europe / Italy / Apulia / Foggia / Stornara
EPI_ISL_1112275	17/02/21	01/03/21	Europe / Italy / Apulia / Foggia / Orta Nova
EPI_ISL_1112276	16/02/21	01/03/21	Europe / Italy / Apulia / Lecce / Lecce
EPI_ISL_1112277	17/02/21	01/03/21	Europe / Italy / Apulia / Lecce / Lecce
EPI_ISL_1112278	18/02/21	01/03/21	Europe / Italy / Apulia / Lecce / Cavallino
EPI_ISL_1112279	16/02/21	01/03/21	Europe / Italy / Apulia / Taranto / Ginosa
EPI_ISL_1112281	17/02/21	01/03/21	Europe / Italy / Apulia / Taranto / Carosino
EPI_ISL_1112282	17/02/21	01/03/21	Europe / Italy / Apulia / Taranto / San Giorgio Ionico
EPI_ISL_1112286	18/02/21	01/03/21	Europe / Italy / Apulia / Taranto / Massafra
EPI_ISL_1112288	18/02/21	01/03/21	Europe / Italy / Apulia / Taranto / Palagiano
EPI_ISL_1112291	17/02/21	01/03/21	Europe / Italy / Apulia / Taranto / Palagiano
EPI_ISL_1116473	01/02/21	02/03/21	Europe / Italy / Apulia / Bari / Bitetto
EPI_ISL_1116474	03/02/21	02/03/21	Europe / Italy / Apulia / Bari
EPI_ISL_1209014	28/02/21	11/03/21	Europe / Italy / Apulia
EPI_ISL_1209020	03/03/21	11/03/21	Europe / Italy / Apulia
EPI_ISL_1209025	04/03/21	11/03/21	Europe / Italy / Apulia
EPI_ISL_1254647	20/01/21	15/03/21	Europe / Italy / Apulia / Lecce / Veglie
EPI_ISL_1254648	07/01/21	15/03/21	Europe / Italy / Apulia / Lecce / Castrignano de' Greci
EPI_ISL_1254649	07/02/21	15/03/21	Europe / Italy / Apulia / Lecce
EPI_ISL_1254650	09/02/21	15/03/21	Europe / Italy / Apulia / Lecce / Cavallino
EPI_ISL_1254651	06/02/21	15/03/21	Europe / Italy / Apulia / Lecce / San Cesario di Lecce
EPI_ISL_1254652	12/02/21	15/03/21	Europe / Italy / Apulia / Foggia

EPI_ISL_1254656	17/02/21	15/03/21	Europe / Italy / Apulia / Foggia / Serracapriola
EPI_ISL_1254658	18/02/21	15/03/21	Europe / Italy / Apulia / Brindisi
EPI_ISL_1254659	18/02/21	15/03/21	Europe / Italy / Apulia / Brindisi / Carovigno
EPI_ISL_1254660	20/02/21	15/03/21	Europe / Italy / Apulia / Brindisi / Fasano
EPI_ISL_1254661	20/02/21	15/03/21	Europe / Italy / Apulia / Brindisi / Fasano
EPI_ISL_1254662	20/02/21	15/03/21	Europe / Italy / Apulia / Brindisi / Fasano
EPI_ISL_1254663	20/02/21	15/03/21	Europe / Italy / Apulia / Brindisi / Mesagne
EPI_ISL_1254664	20/02/21	15/03/21	Europe / Italy / Apulia / Brindisi / Fasano
EPI_ISL_1254665	22/02/21	15/03/21	Europe / Italy / Apulia / Lecce / Calimera
EPI_ISL_1254667	09/02/21	15/03/21	Europe / Italy / Apulia / Lecce / Cavallino
EPI_ISL_1254668	17/02/21	15/03/21	Europe / Italy / Apulia / Lecce
EPI_ISL_1254669	20/02/21	15/03/21	Europe / Italy / Apulia / Lecce / Otranto
EPI_ISL_1254670	22/02/21	15/03/21	Europe / Italy / Apulia / Lecce
EPI_ISL_1254671	23/02/21	15/03/21	Europe / Italy / Apulia / Foggia / Chieuti
EPI_ISL_1254675	25/02/21	15/03/21	Europe / Italy / Apulia / Brindisi / Fasano
EPI_ISL_1254676	01/03/21	15/03/21	Europe / Italy / Apulia / Brindisi
EPI_ISL_1254677	01/03/21	15/03/21	Europe / Italy / Apulia / Brindisi / Fasano
EPI_ISL_1254678	01/03/21	15/03/21	Europe / Italy / Apulia / Brindisi / Torchiarolo
EPI_ISL_1254679	01/03/21	15/03/21	Europe / Italy / Apulia / Brindisi / Fasano
EPI_ISL_1254681	01/03/21	15/03/21	Europe / Italy / Apulia / Bari / Sannicandro di Bari
EPI_ISL_1254682	25/02/21	15/03/21	Europe / Italy / Apulia / Bari / Acquaviva delle Fonti
EPI_ISL_1254683	01/03/21	15/03/21	Europe / Italy / Apulia / Bari / Acquaviva delle Fonti
EPI_ISL_1254684	01/03/21	15/03/21	Europe / Italy / Apulia / Bari / Adelfia
EPI_ISL_1254685	01/03/21	15/03/21	Europe / Italy / Apulia / Bari / Acquaviva delle Fonti
EPI_ISL_1254686	01/03/21	15/03/21	Europe / Italy / Apulia / Bari / Acquaviva delle Fonti
EPI_ISL_1254687	01/03/21	15/03/21	Europe / Italy / Apulia / Bari / Acquaviva delle Fonti
EPI_ISL_1254690	20/02/21	15/03/21	Europe / Italy / Apulia / Foggia
EPI_ISL_1254691	20/02/21	15/03/21	Europe / Italy / Apulia / Foggia
EPI_ISL_1254692	02/03/21	15/03/21	Europe / Italy / Apulia / Bari
EPI_ISL_1254694	27/02/21	15/03/21	Europe / Italy / Apulia / Foggia / Serracapriola
EPI_ISL_1254696	03/03/21	15/03/21	Europe / Italy / Apulia / Taranto
EPI_ISL_1254697	03/03/21	15/03/21	Europe / Italy / Apulia / Taranto
EPI_ISL_1254698	03/03/21	15/03/21	Europe / Italy / Apulia / Taranto
EPI_ISL_1254699	26/02/21	15/03/21	Europe / Italy / Apulia / Taranto / Massafra
EPI_ISL_1254700	27/02/21	15/03/21	Europe / Italy / Apulia / Taranto / San Giorgio Ionico
EPI_ISL_1254701	04/03/21	15/03/21	Europe / Italy / Apulia / Lecce / Miggiano
EPI_ISL_1254702	28/02/21	15/03/21	Europe / Italy / Apulia / Foggia / San Giovanni Rotondo
EPI_ISL_1254703	28/02/21	15/03/21	Europe / Italy / Apulia / Foggia / San Giovanni Rotondo
EPI_ISL_1261868	12/02/21	16/03/21	Europe / Italy / Apulia / Lecce / Cavallino
EPI_ISL_1261869	01/03/21	16/03/21	Europe / Italy / Apulia / Bari / Casamassima
EPI_ISL_1261870	02/03/21	16/03/21	Europe / Italy / Apulia / Bari / Bitetto
EPI_ISL_1391021	09/03/21	29/03/21	Europe / Italy / Apulia / Bari
EPI_ISL_1391023	08/03/21	29/03/21	Europe / Italy / Apulia / Bari / Acquaviva delle Fonti
EPI_ISL_1416331	16/02/21	31/03/21	Europe / Italy / Apulia / Foggia
EPI_ISL_1417440	03/03/21	31/03/21	Europe / Italy / Apulia / Foggia

EPI_ISL_767013	27/12/20	05/01/21	Europe / Italy / Apulia
EPI_ISL_794747	23/12/20	11/01/21	Europe / Italy / Apulia / Lecce
EPI_ISL_794748	24/12/20	11/01/21	Europe / Italy / Apulia / Bari
EPI_ISL_794750	22/12/20	11/01/21	Europe / Italy / Apulia / Lecce
EPI_ISL_794751	23/12/20	11/01/21	Europe / Italy / Apulia / Lecce
EPI_ISL_826458	29/12/20	15/01/21	Europe / Italy / Apulia
EPI_ISL_918408	27/12/20	03/02/21	Europe / Italy / Apulia / Lecce / Lizzanello
EPI_ISL_918409	29/12/20	03/02/21	Europe / Italy / Apulia / Lecce / Carmiano
EPI_ISL_918410	23/01/21	03/02/21	Europe / Italy / Apulia / Bari
EPI_ISL_940565	18/01/21	05/02/21	Europe / Italy / Apulia
EPI_ISL_940631	18/01/21	05/02/21	Europe / Italy / Apulia
EPI_ISL_940632	19/01/21	05/02/21	Europe / Italy / Apulia
EPI_ISL_940739	25/01/21	05/02/21	Europe / Italy / Apulia
EPI_ISL_949182	18/01/21	08/02/21	Europe / Italy / Apulia
EPI_ISL_949184	27/01/21	08/02/21	Europe / Italy / Apulia
EPI_ISL_949185	27/01/21	08/02/21	Europe / Italy / Apulia
EPI_ISL_949188	20/01/21	08/02/21	Europe / Italy / Apulia
EPI_ISL_949191	27/01/21	08/02/21	Europe / Italy / Apulia
EPI_ISL_977495	03/02/21	11/02/21	Europe / Italy / Apulia
EPI_ISL_977496	04/02/21	11/02/21	Europe / Italy / Apulia
EPI_ISL_977497	05/02/21	11/02/21	Europe / Italy / Apulia
EPI_ISL_977498	04/02/21	11/02/21	Europe / Italy / Apulia
